# Datasets of drought and flood impact on croplands in Southeast Asia from 1980 to 2019

**DOI:** 10.1016/j.dib.2021.107406

**Published:** 2021-09-20

**Authors:** Manjunatha Venkatappa, Nophea Sasaki

**Affiliations:** a LEET Intelligence Co., Ltd., Suan Prikthai, Muang Pathum Thani, Pathum Thani 12000, Thailand; b Natural Resources Management, SERD, Asian Institute of Technology. P.O. Box 4, Khlong Luang, Pathum Thani 12120, Thailand

**Keywords:** Climate change, Google earth engine, Agriculture, Drought, Crop damage, Gridded datasets

## Abstract

Data on droughts and floods and their impacts on croplands and production are important for policy makers and the scientific community. This dataset was developed to provide data of the impacts of droughts and floods on agriculture in the Monsoon Climate Region and Equatorial Climate Region of Southeast Asia during the crop growing seasons over a 40-year period between 1980 and 2019. The data were generated using the TerraClimate global high-resolution gridded Palmer Drought Severity Index (PDSI) datasets in Google Earth Engine along with a set of algorithms. Datasets were available on 47,192 grid points of a 10 × 10 km resolution containing PDSI, their latitude longitude between 1980 and 2019 with five years interval, monthly temporal PDSI data, cropland drought and flood intensity data between 1980 and 2019.

## Graphical abstract

**Figure:**
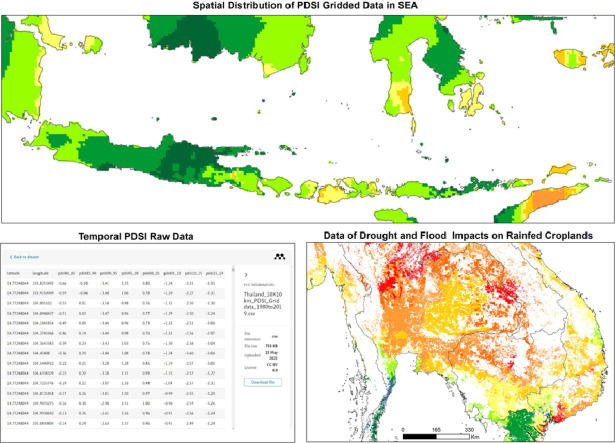

## Specifications Table

**Table:** 

Subject	Environmental Science (General)
Specific subject area	Drought and flood impact on croplands in Southeast Asia
Type of data	Table Figure Maps
How data were acquired	The Palmer Drought Severity Index (PDSI) data and cropland data were collected in Google Earth Engine (GEE) for the entire Southeast Asia (SEA). PDSI monthly time series data was created during the crop-growing seasons between 1980 and 2019. The PDSI gridded data were generated at 10 km × 10 km in SEA.
Data format	Raw data available in Comma Separated Values (CSV) files Gridded data available in CSV files Processed data available in tables Cropland data available in ArcGIS shapefiles Map data available in .jpg format
Parameters for data collection	The PDSI data were examined in GEE during the main crop-growing seasons, May to November for the Monsoon Climate Region (MCR) and October to April for the Equatorial Climate Region (ECR) in SEA from 1980 to 2019. We generated 47,192 (10 km × 10 km) spatial gridded data, which were used to identify the frequency distribution of the PDSI and change in intensity of the drought events as measured by the frequencies of occurrence from 1980 to 2019. Only rainfed cropland data by combining three rainfed crops (rainfed, rainfed minor fragments, rainfed very minor fragments) to assess droughts and floods damage.
Description of data collection	PDSI datasets were acquired in Google Earth Engine (GEE) during the main crop-growing seasons, May to November for the MCR (Myanmar, Thailand, Lao PDR, Cambodia, and Vietnam) and October to April for the ECR (Malaysia, Singapore, Indonesia, the Philippines, and Brunei Darussalam) between 1980 and 2019. Regional level seasonal PDSI profiles were generated by country using Microsoft excel. Further, to assess the drought conditions and their impact on croplands and crop production in SEA, we generated 47,192 (10 km × 10 km) geographic grid points. The Global Food Security-support Analysis (GFSAD) cropland data was used to assess the damages caused by droughts and floods on the rainfed croplands in the MCR and ECR in SEA. Any cropland is considered damaged when the PDSI was less than -2.00 (corresponding to moderate drought) or when the PDSI is greater than 2.00 (moderately wet) during the crop-growing seasons in the MCR and ECR, respectively. For -1.00 < PDSI ≤ 1.00, no damage was considered.
Data source location	Indonesia, Vietnam, Thailand, Philippines, Singapore, Malaysia, Myanmar (Burma), Laos PDR, Cambodia, and Brunei https://data.mendeley.com/datasets/nv7jbmkynm/3
Data accessibility	Repository name: Mendeley repository • Data identification number: DOI: 10.17632/nv7jbmkynm.3 • Direct URL to data: https://data.mendeley.com/datasets/nv7jbmkynm/3
Related research article	Manjunatha Venkatappa, Nophea Sasaki, Han Phoumin, Issei Abed, Impacts of droughts and floods on croplands and crop production in Southeast Asia – An Application of Google Earth Engine, *Science of The Total Environment*, Volume 795, 2021, 148829, ISSN 0048-9697, https://doi.org/10.1016/j.scitotenv.2021.148829.

## Value of the Data

• Long-term data of the spatial impacts of climate-driven droughts and floods are urgently needed to aid the better-informed decision making, which can reduce the crop damages spatially and temporally at scale.
• Data on the intensity of climate change at 10 km resolution across the region can be useful for validating the results from the regional climate models for improving the accuracy of the prediction of future climate change and its impacts on croplands.
• Data of PDSI over the last 40 years for the whole Southeast Asia can be further used for education, training, and capacity building in climate change impact assessment and prevention measures across scale.

## 1 Data Description

In this data brief we described four datasets including, two raw data, Geographic Information System (GIS) shapefile data, and processed data in tables and maps in supplementary materials on drought and its impact on rainfed agriculture lands in the Monsoon Climate Region (MCR) and Equatorial Climate Region (ECR) of Southeast Asia (SEA). The first raw dataset (Table 1) contains monthly temporal Palmer drought Severity index (PDSI) [1] by country between 1980 and 2019, while the second raw gridded point dataset (Table 2) covers average drought frequencies by 10 km × 10 km geographically in MCR and ECR regions with five years interval between 1980 and 2019 [2]. The third data in GIS Shapefile format (Fig. 4) comprehends drought and their severity levels on rainfed croplands by country in SEA in 2010 [3]. The fourth data in tables contain analyzed average of gridded PDSI values during the crop-growing season from May to November for the MCR (Myanmar, Thailand, Lao PDR, Cambodia, and Vietnam) and October to April for the ECR (Malaysia, Singapore, Indonesia, the Philippines, and Brunei Darussalam) with five years interval between 1980 and 2019 [2] and crop production loss due to drought and floods by country. The maps data which represents severity classes of drought and wet conditions (floods) impact on rainfed croplands, and the level of need for policy interventions by country in the MCR and ECR countries in SEA [2]. All data including raw data, processed data, GIS cropland data and maps were stored in a specific country folder (https://data.mendeley.com/datasets/nv7jbmkynm/3).

**Fig. 1:**
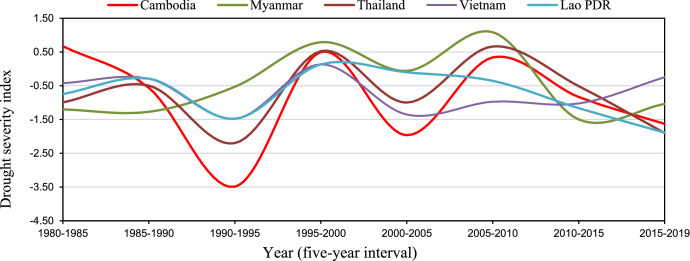
Average drought condition of five-year intervals during the crop growing season in MCR countries.

Source: Authors.

**Fig. 2:**
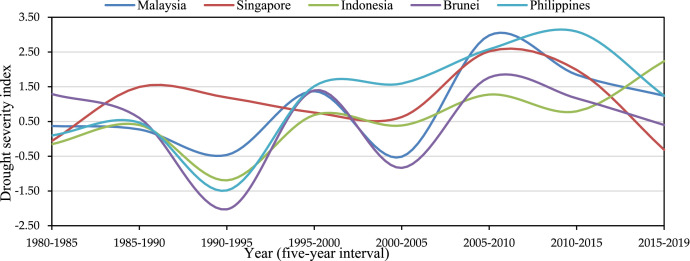
Temporal drought severity index by country (five-year interval) during the crop growing season (October–April) in the ECR.

Source: Authors.

**Fig. 3:**
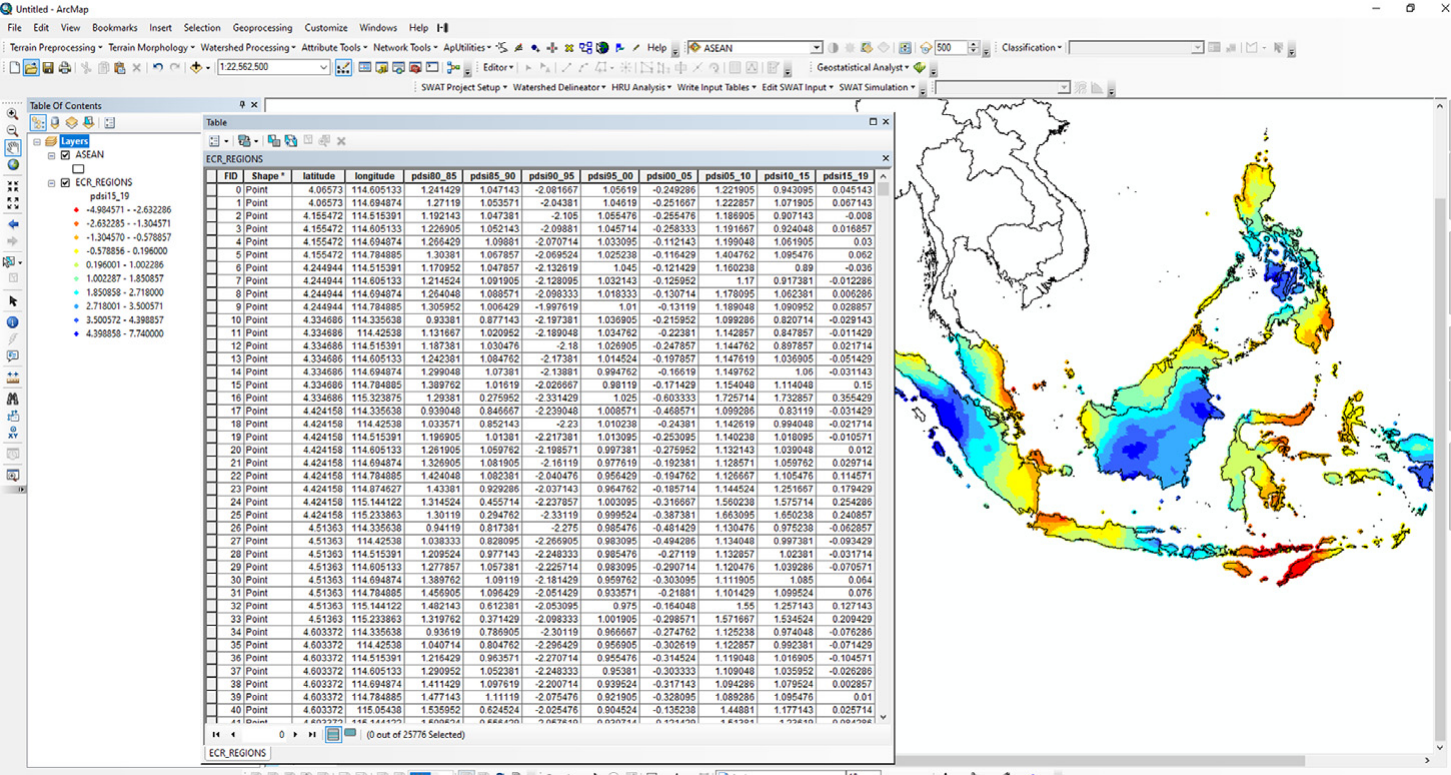
Example screenshot of spatial distribution of drought and wet frequencies detected in ECR during the crop-growing seasons is displayed in the Esri ArcMap software. The dot color palette represents the PDSI levels. PDSI = Palmer Drought Severity Index

Source: Authors.

• Raw dataset: Raw data Table 1 file provides temporal PDSI data monthly between 1980 and 2019 in CSV file. The data file attribute includes system: time_start (Time: PDSI data by monthly from 1980 to 2019), PDSI (Palmer drought Severity index values), and the Average PDSI (PDIS values by yearly) (Table 1). Figs. 1 and 2, presents examples of temporal average drought conditions of five-year intervals during the crop growing season in MCR (Fig. 1) and ECR (Fig. 2) in SEA, which were derived from the datasets.

• Gridded dataset: The 10 km × 10 km gridded points CSV file in SEA provides drought intensities during the crop growing season (Table 2). The file includes location with latitude, longitude, average PDSI with five years intervals between 1980 and 2019: pdsi1980_1985, pdsi1985_1990, pdsi1990_1995, pdsi1995_2000, pdsi200_2005, pdsi2005_2010, pdsi2010_2015 and pdsi2015_2019. Using the location-based grid data can assess the spatial pattern of PDIS frequencies distribution in a particular country during the crop growing season (Fig. 3) [2]. Table 2 represents example of total a number of the 10 km × 10 km grid points covered by country in the SEA in the datasets.

• GIS dataset: The rainfed cropland ArcGIS shapefile data derived from the Global Food Security-support Analysis (GFSAD) 1,000 nominal cropland class: rainfed, rainfed minor fragments and rainfed very minor fragments [3] were selected to assess droughts and floods damage and crop production loss in the MCR and ECR during the crop-growing seasons (Fig. 5). The cropland dataset stored in ArcGIS shapefile format and the data geographical reference system presented as below:
  - Geographic Coordinate System: GCS_WGS_1984
  - Datum: D_WGS_1984
  - Prime Meridian: Greenwich
  - Angular Unit: Degree

**Table 1:** Example of monthly and yearly temporal raw data of PDSI in the CSV file.

system:			Average	system:			Average
time_start	PDSI	Year	PDSI	time_start	PDSI	Year	PDSI
1-Jan-80	-4.27	1980	-1.22	1-Sep-81	-0.71	2000	5.22
1-Feb-80	-4.70	1981	0.32	1-Oct-81	-1.17	2001	0.63
1-Mar-80	-4.78	1982	-0.72	1-Nov-81	0.02	2002	-1.20
1-Apr-80	-4.37	1983	0.67	1-Dec-81	-0.16	2003	-0.69
1-May-80	-3.67	1984	2.63	1-Jan-82	0.10	2004	-2.80
1-Jun-80	-2.60	1985	2.34	1-Feb-82	0.22	2005	-5.76
1-Jul-80	-1.66	1986	-0.13	1-Mar-82	0.47	2006	0.40
1-Aug-80	-0.87	1987	-1.77	1-Apr-82	0.28	2007	3.48
1-Sep-80	-0.08	1988	2.19	1-May-82	-1.09	2008	-0.36
1-Oct-80	0.10	1989	-1.86	1-Jun-82	-0.52	2009	0.94
1-Nov-80	0.21	1990	-1.25	1-Jul-82	-1.01	2010	-2.78
1-Dec-80	0.10	1991	-2.70	1-Aug-82	-1.27	2011	0.66
1-Jan-81	0.16	1992	-4.71	1-Sep-82	-0.32	2012	-1.09
1-Feb-81	1.17	1993	-5.82	1-Oct-82	-0.69	2013	-0.18
1-Mar-81	0.63	1994	-1.91	1-Nov-82	-0.15	2014	0.59
1-Apr-81	0.42	1995	-2.30	1-Dec-82	0.00	2015	-4.15
1-May-81	0.61	1996	-0.81	1-Jan-83	0.08	2016	-5.35
1-Jun-81	1.29	1997	-0.66	1-Feb-83	-0.11	2017	0.80
1-Jul-81	1.27	1998	-4.25	1-Mar-83	-0.90	2018	1.57
1-Aug-81	0.95	1999	2.97	1-Apr-83	-2.36	2019	-3.54

Source: Authors

**Table 2:** Example of 10 km × 10 km spatial gridded CSV dataset of PDSI during the crop growing season from 1980–2019 is in the Raw data folder in Mendeley experiment data files.

		Pdsi	Pdsi	Pdsi	Pdsi	Pdsi	Pdsi	Pdsi	Pdsi
latitude	longitude	80_85	85_90	90_95	95_00	00_05	05_10	10_15	15_19
11.53639	104.9033	1.45	0.01	-2.58	-1.27	-1.81	-0.49	-0.81	-1.04
11.53639	104.9931	1.58	0.05	-2.81	-1.42	-2.03	-1.03	-1.03	-1.31
11.53639	105.0831	1.58	0.08	-3.20	-1.45	-2.10	-1.40	-1.05	-1.34
11.53639	105.1728	1.56	0.12	-3.35	-1.44	-2.04	-0.93	-1.13	-1.40
11.53639	105.2626	1.53	0.14	-3.41	-1.29	-1.91	-0.91	-1.19	-1.31
11.53639	105.3526	1.50	0.13	-3.42	-1.22	-1.89	-0.93	-1.20	-1.32
11.53639	105.4423	1.50	0.07	-3.50	-1.17	-1.86	-0.90	-1.22	-1.28
11.53639	105.5321	1.54	0.08	-3.64	-1.17	-2.00	-1.03	-1.27	-1.39
11.53639	105.6221	1.53	0.03	-3.84	-1.14	-2.09	-1.44	-1.30	-1.46
11.53639	105.7118	1.50	0.02	-4.09	-1.09	-2.17	-1.34	-1.41	-1.61
11.53639	105.8016	1.13	0.00	-4.07	-0.90	-1.88	-0.54	-1.21	-1.64
11.62424	103.1066	-0.54	0.91	-2.78	-0.41	-0.71	1.41	1.35	0.46
11.62424	103.1966	-0.46	0.93	-2.84	-0.47	-0.87	1.33	1.30	0.38
11.62424	103.2864	-0.16	0.73	-2.76	-0.44	-0.93	1.27	1.12	0.12

Source: Authors

**Fig. 4:**
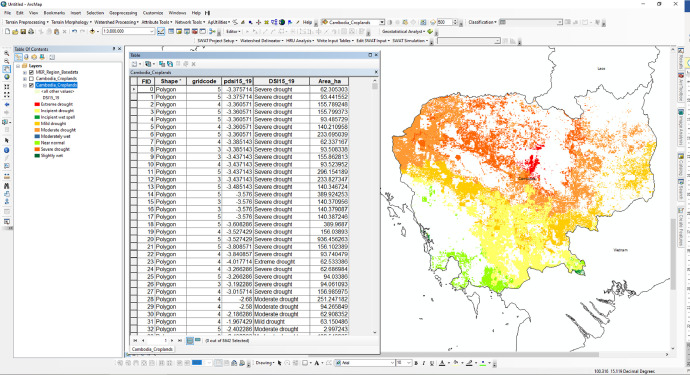
Example screenshot of rainfed cropland ArcGIS shapefile dataset attribute in Cambodia is displayed in the Esri ArcMap software.

Source: Authors.

The rainfed cropland ArcGIS shapefile (Fig. 4) data description includes:

1. FID: Unique identifier of cropland polygon features
2. Shape: Cropland polygon feature
3. gridcode: Rainfed cropland class code: gridcode 3 = Rainfed cropland class, gridcode 4 = Rainfed minor fragments and gridcode 5 = Rainfed very minor fragments croplands
4. PDIS2019: Cropland average PDSI values between 2015 and 2019,
5. DSI2019: Cropland Drought severity class
6. Area_ha: Rainfed cropland area affected by drought and flood severity.

### 1.1 Dataset access and process

**Fig. 5:**
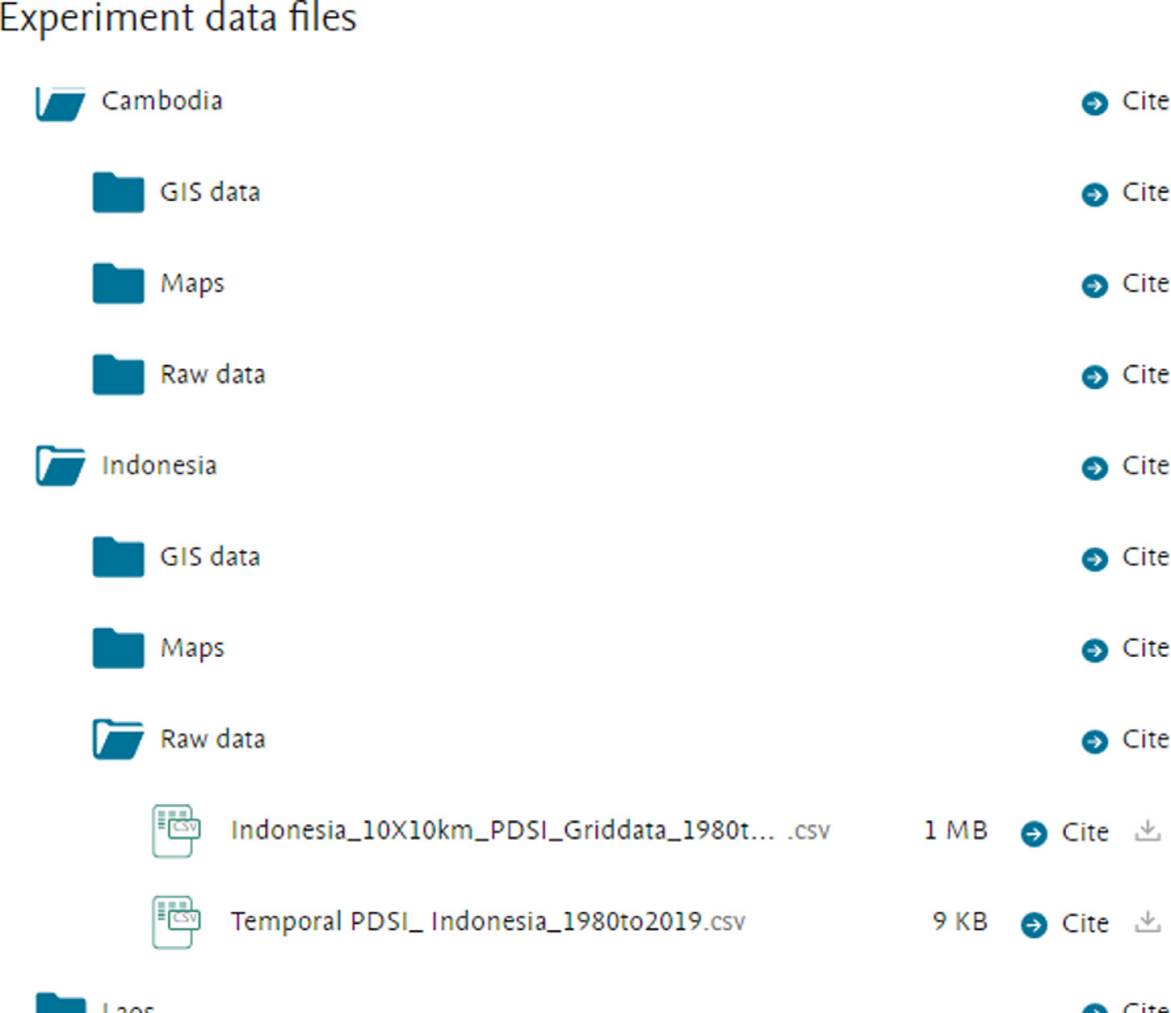
Example screenshot of Mendeley experiment data folder.

All data including, raw data, processed data, ArcGIS shapefiles and maps were stored in Mendeley data by country can be accessed freely (https://data.mendeley.com/datasets/nv7jbmkynm/3). Each country folder includes 3 subfolders: GIS data, Maps, and Raw data (Fig. 5).

The data users: researchers, NGOs, development agencies, and policymakers can download the data by using below methods.

ArcMap and QGIS users:

1. Download raw data files.
2. Assess the temporal drought and flood conditions using Microsoft Excel or any relevant software that supports reading the CSV files.
3. Convert gridded data CSV file to ArcGIS shapefiles by using ArcGIS X (longitude) Y (latitude) using the Table to Point in the Data Management tool or use the QGIS by adding an X and Y coordinate to Point Data using the Add Delimited Text Layer tool.
4. Use Interpolation or suitable spatial analysis methods in ArcGIS or Qgis to analyze the drought intensities by country.
5. Download rainfed cropland GIS shapefile files. Overly cropland data on gridded spatial data and assess drought and flood impacts on rainfed croplands in SEA.

Google Earth Engine users:

Use the Asset Manager in the Code Editor to upload CSV datasets. The Asset Manager is on the Assets tab at the left side of the Code Editor. Use the Importing Table Data to upload the CSV table data into GEE. You can import an asset (CSV data) to your script in Code Editor by hovering over the asset name in the Asset Manager [4].

## 2 Experimental Design, Materials and Methods

### 2.1 Study area

**Fig. 6:**
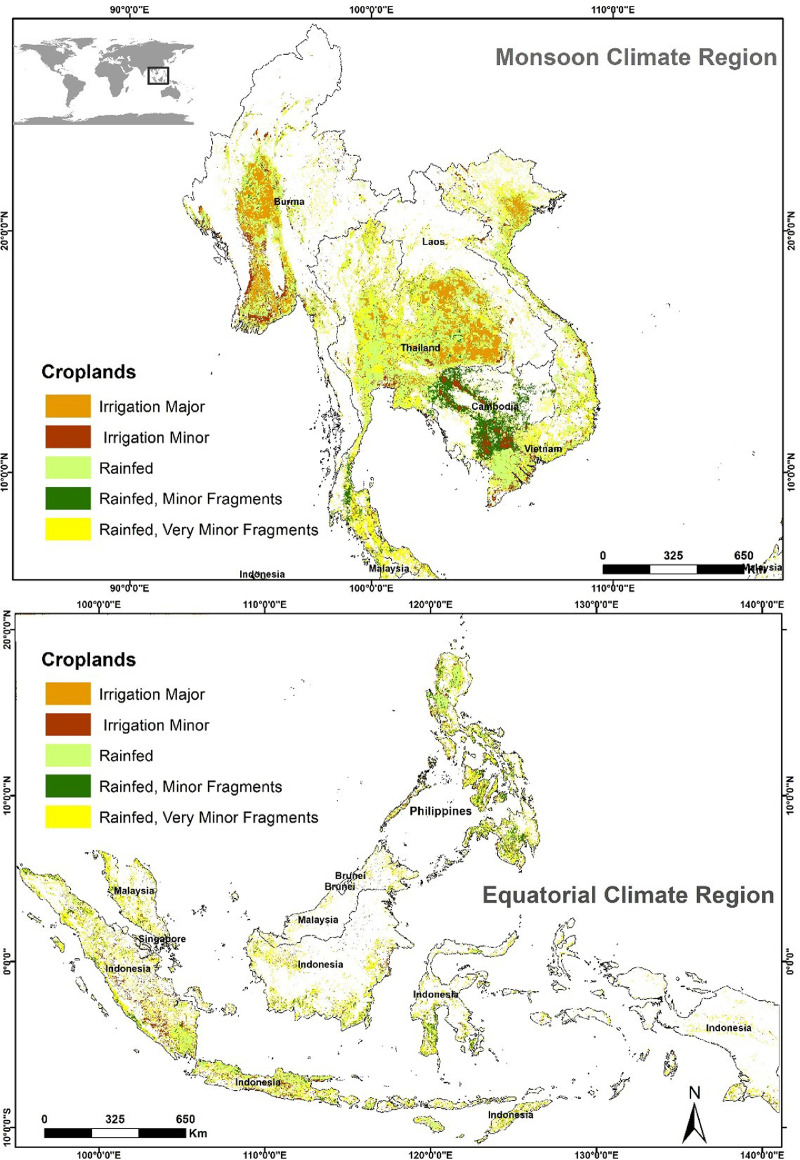
Maps showing the locations of the Southeast Asia Monsoon Climatic Region and Equatorial Climatic Region Note: The Fig. 6 was modified from Venkatappa et al., 2021 [5]. Lao = Lao People's Democratic Republic, Brunei = Brunei Darussalam.

Source: Authors.

Southeast Asia was divided into two regions, namely the MCR and ECR, because of their distinct climate variabilities, creating differences in rainfall, temperature, and crop planting seasons. The primary crop-growing seasons are May–November in the MCR and October–April in the ECR [5]. The MCR is composed of Myanmar, Thailand, Lao PDR, Cambodia, and Vietnam, and the ECR of Malaysia, Singapore, Indonesia, the Philippines, and Brunei Darussalam (Fig. 6).

### 2.2 Data of PDSI on croplands

The PDSI data were examined using the GEE and ArcMap during the main crop-growing seasons, May to November for the MCR and October to April for the ECR over the 40 years from 1980 to 2019. Here, dryness refers to drought conditions and wetness refers to floods as defined by the values of PDSI [1]. JavaScript [4] programming language was used in the GEE to collect the PDSI monthly time series data during the crop-growing seasons in the SEA region. The generated PDSI profiles by country were computed in the GEE and then exported outside the GEE for generating seasonal PDSI profiles using Microsoft Excel. Subsequently, temporal PDSI values in the MCR and ECR were assessed during the crop-growing seasons, monthly, yearly, and with five-year intervals between 1980 and 2019.

Further, to assess the drought conditions and their impact on croplands and crop production in SEA, we generated 47,192 (10 km × 10 km) spatial grid data, which were used to identify the frequency distribution of the PDSI and change in intensity of the drought events as measured by the frequencies of occurrence over the 40-year period of this study. The PDSI values were extracted into grid point data using the value extraction function by country from 2015 to 2019 in the GEE. The gridded point data were then applied by country and the PDSI values were extracted into the gridded points. Crop damage levels were established to trigger the drought- and flood-caused damages to the croplands. Any cropland is considered damaged when the PDSI was less than -2.00 (corresponding to moderate drought) or when the PDSI is greater than 2.00 (moderately wet) during the crop-growing seasons in the MCR and ECR, respectively. For -1.00 < PDSI ≤ 1.00, no damage was considered [5].

### 2.3 Assessment of drought and floods damages on croplands and crop production

The GFSAD 1000 nominal cropland was classified into seven classes, namely water, irrigation major, irrigation minor, rainfed, rainfed minor fragments, rainfed very minor fragments and non-croplands [3]. Here we combined three rainfed crops (rainfed, rainfed minor fragments, rainfed very minor fragments) data [3] to assess the droughts and floods damage and crop production [6] loss in the MCR and ECR (Fig. 6). The irrigated croplands were not included in our data assessment because we focused on the impacts of droughts and floods on rainfed croplands and crop production during the crop-growing seasons. Since rice is the major crop in SEA, rice was assumed to be the rainfed crop whose lands and production were damaged by the droughts and floods for assessing the drought and flood damages on crop in this study. As we analyzed the drought and floods only in the growing seasons (i.e., in the rainy season), only rainfed crops were covered. Damages caused by droughts and floods on the rainfed croplands, and their production loss were assessed for the MCR and ECR. PDSI attributes values were assigned to the rainfed croplands by using Spatial join tool in Esri ArcMap based on spatial relationship and the drought and flood conditions were assessed by area over the past five years only from 2015 to 2019. Crop damage levels [5] were established to trigger the drought- and flood-caused damages to the croplands [1].

The croplands and damages to crop production were assessed on the 10 km × 10 km gridded spatial point data across the MCR and ECR regions. The percentage of cropland damages, and the number of people affected by droughts were then calculated by country in SEA by applying Eqs. (1) to (3).

*Total area of crop damage:*

(1) $$TCDA_{k}=\;\sum_{k=1}^{1}\sum_{i=1}^{6}CD_{ik}$$

where:

$TCDA_{k}$= Total crop damaged area in country *k* in Southeast Asia (ha year^−1^)

$CD_{ik}$ = Crop damage corresponding to crop damage levels (6) *i* in country *k* was obtained by using the GEE (ha year^−1^).

*Crop production loss:*

(2) $$TCPl_{k}=\;\sum_{i=1}^{6}\left(CD_{ik}\times CR_{ik}\times CP_{k}\right)$$

where:

$TCPl_{k}$ = Total crop production lost or damaged by drought or wet in country *k* (tons year^−1^)

$CR_{ik\;}$ = Crop production reduction (proportion of crop damage) by crop damage level *i* (%)

As rice is the main diet in Southeast Asia, assessment of the people affected by such droughts and floods could provide useful information for policymakers to prioritize appropriate interventions. The loss of crop production was estimated using rice production to represent rainfed crops in the SEA region [6] (ton yr^−1^) during the period 2015–2019 by applying the effects of drought and flood stress on rice yield (CR_ik_ in our study). Under the dry condition, crop production reduction CR_ik_ was estimated at 27.8% of rice yield for a moderate drought, 32.0% for a severe drought, and 90.0% or almost 100% for extreme drought conditions [7]. Crop production reduction because of floods was calculated at 16.6% for a moderate wet condition, 22.5% for a very wet condition, and 33.3% for wet condition. We used all these reduction rates for Eq. (2) [5].

*People affected by crop production damage:*

To estimate the effects of crop production loss on population, the following equation was used.

(3) $$PA_{k}=\frac{TCPl_{i}}{PC}$$

where:

$PA_{k}$ = People affected by crop production damage by country *k*

*PC* = Per-capita rice (crop) consumption. It is 200 km (0.2 tons) of rice per person per year [8].

## CRediT authorship contribution statement

**Manjunatha Venkatappa:** Conceptualization, Methodology, Software, Data curation, Writing – original draft, Visualization, Investigation. **Nophea Sasaki:** Methodology, Writing – review & editing.

## Declaration of Competing Interest

The authors declare that they have no known competing financial interests or personal relationships that could have appeared to influence the work and data reported in this article.
